# ‘We are doing damage control’: Government stakeholder perspectives of educational and other services for children with autism spectrum disorder in South Africa

**DOI:** 10.1177/13623613221142111

**Published:** 2022-12-13

**Authors:** Sarosha Pillay, Madeleine Duncan, Petrus J de Vries

**Affiliations:** University of Cape Town, South Africa

**Keywords:** autism spectrum disorder, education, government stakeholder perspectives, low- and middle-income countries, qualitative research, service delivery

## Abstract

Autism spectrum disorder is a growing public health concern in low-and middle-income countries that has prompted a World Health Organization resolution on ‘*comprehensive and coordinated efforts for management of autism spectrum disorder’*. Despite being a signatory of the resolution, South Africa does not have any national policies to guide the management of autism spectrum disorder services. This study explored the perspectives of key government stakeholders on educational and other services for children with autism spectrum disorder in the Western Cape Province of South Africa and their proposed solutions to meet the needs of autistic children and their families. The overarching theme that emerged was ‘We are doing damage control’. Government stakeholders acknowledged that autism spectrum disorder services were falling between the ‘cracks’ caused by competing societal demands on government resources. Participants shared perspectives about autism spectrum disorder services in three categories: *a cracked society, siloed service systems* and *gap-filling strategies*. Findings from this study highlighted the need for collaborative efforts between government departments and civil society to develop a strategy for autism spectrum disorder in line with the World Health Organization resolution. Multistakeholder engagement to break down barriers, strengthen systems and develop innovative solutions to improving services for children with autism spectrum disorder and their families is recommended.

**Lay abstract:**

Autism spectrum disorder is a growing public health concern in low- and middle-income countries like South Africa where there are no plans or policies in place for autism spectrum disorder management. Many children with autism spectrum disorder in South Africa are out of schools and waiting for school placement to become available. This study explored the perspectives of key government stakeholders on educational and other services for children with autism spectrum disorder in the Western Cape Province of South Africa and their suggestion for improving services for these children and their families. Semi-structured interviews were conducted with government stakeholders from the Western Cape Department of Education, Department of Health and the Department of Social development. The main theme that emerged was ‘We are doing damage control’. Government stakeholders acknowledged that autism spectrum disorder services were being overlooked because of other demands on government resources. Finding from this study highlighted the need for government departments to work together to develop a strategy for autism spectrum disorder management. Engagement between government and civil society to break down barriers, strengthen systems and develop solutions to improving access to services for children with autism spectrum disorder and their families is recommended.

## Introduction

Although South Africa is classified as an upper-middle income country (The World Bank, 2022), more than half of the population live below the upper-bound poverty line (currently ZAR1227/USD87 per person per month) (Sulla & Zikhali, 2018). With one of the highest Gini coefficients globally, the economic divide between rich and poor in South Africa is starkly visible in the unmet health and social needs of a large percentage of the population. Disparities in access to housing, sanitation, nutrition and education services between rich and poor are attributed to a growing quadruple public health burden of disease (maternal, new-born and child health; HIV/AIDS and tuberculosis; non-communicable diseases; and violence and injury) intersecting with inadequate human and infrastructure resources (Bradshaw et al., 2019; Sulla & Zikhali, 2018).

Education is fundamental for the social and economic growth and development of nations (Shrivastava & Shrivastava, 2014; United Nations General Assembly, 2015). Development in the South African education system requires the complex socio-economic legacies of the apartheid era that excluded children from non ‘White’ racial groups and children with disabilities from appropriate educational settings to be addressed (Bornman & Donohue, 2014; Engelbrecht, 2020). In correcting the injustices of the past, the education system is working towards the full participation in education for all children including those with diverse learning needs (Department of Education, 2001, 2007, 2014). To ensure equity and social justice for all citizens, including those with disability, the post-apartheid South African education system adopted national policies that align with the United Nations Convention on the Rights of the Child (1998, Article 23) and the Convention on the Rights of Persons with Disabilities (2006, Article 24) (The United Nations, 2006; United Nations General Assembly, 1989). The main components of these policies are that every child irrespective of ability has the right to an inclusive education system, the right to accessible education, and the right to individualised support (Hodgson, 2018).

Well-developed policies provide a framework for service delivery and monitoring, including the government’s commitment to and accountability for funding and resource allocation to enable implementation (Mokitimi et al., 2018). Education policy enactment in South Africa has been problematic due to contextual factors, policy weaknesses and poor guidelines for implementation (Engelbrecht, 2020). Policy-aligned education for children with disability is inadequate and specific policies for children with diverse learning needs such as those with autism spectrum disorder (ASD) have not yet been developed. For example, an estimated 597,953 South African children with disabilities were reported to be out of school (Department of Basic Education, 2015). While it is unknown how many of these children have ASD, there are no ASD specific policies or guidelines for service delivery including ASD education services in any Sub-Saharan African country (de Vries, 2016).

With an estimated 78 million autistic people worldwide, ASD is rapidly becoming a global public health concern due to the significant impact of the diagnosis on individuals, families and society (Lord et al., 2022). The burden of disease and associated socio-economic costs of ASD is widely reported particularly in low- and middle-income countries (LMIC) (Baxter et al., 2015; Clasquin-Johnson & Clasquin-Johnson, 2018; Leigh & Du, 2015; World Health Organization (WHO), 2014) and the majority of people with ASD do not have access to services (Lord et al., 2022). In South Africa, there is an urgent need for attention to policy design and implementation (de Vries, 2016; Franz et al., 2017, 2018; Pillay et al., 2021, 2022). In 2014, the World Health Assembly resolution WHA67.8, ‘Comprehensive and coordinated efforts for management of autism spectrum disorder’ called for government actions to improve the quality of life of individuals with ASD and their families (WHO, 2014). It urged governments to develop new ASD policies or update existing policies that are aligned with the WHA67.8 and to have multisectoral strategies for implementation of these policies (WHO, 2014). Despite the adoption of this resolution, information on ASD in South Africa and other LMIC remains limited (Bakare et al., 2019; de Vries, 2016; Tekola et al., 2016) and there is no strategic plan for identification, management and education of children with ASD in South Africa.

The *Lancet* Commission on the future of care and clinical research in autism (Lord et al., 2022) found that ASD knowledge at government and policy development levels is generally poor. ASD services are is not prioritised in some LMIC due to other competing socio-economic and public health challenges that these governments are faced with (Hahler & Elsabbagh, 2015). Franz and colleagues (2018) interviewed senior management-level government stakeholders in the Western Cape to examine their perspectives on early detection and intervention for children with ASD in South Africa. They found that most of the government stakeholders acknowledged that ASD was an area of growing concern; however, early detection and intervention was not supported by policy, goals and values of all government departments. They suggested that more local information on ASD was necessary for better management and support for these children.

Recent work by Pillay et al. (2021) on educational services for children with ASD in the Western Cape Province of South Africa identified poor systems for identification and reporting of children with ASD, a lack of essential early intervention programmes, and education system failure to accommodate children with ASD in appropriate educational settings. In a separate study by Pillay et al. (2022), a total of 744 children with ASD or suspected ASD were reported to be waiting for special school placement over the same period of which 36% (*n* = 265) were of compulsory school-going age. Children with ASD being out of school is an infringement of their constitutional rights. Acknowledging the complexities associated with disability education, a whole-system understanding is indicated to determine why this violation of human rights is happening.

In health systems research, Gilson and colleagues (2017) recognise governance as a complex process with multiple layers that influence decision-making, policy enactment and service delivery. Therefore, an understanding of the ‘hardware’ (e.g. finances, resources and infrastructure) and ‘software’ (e.g. values, norms and relationships) elements of a system is necessary for ‘whole-systems’ understanding (Gilson et al., 2017). In examination of the ‘hardware’ of education systems for children with ASD in the Western Cape, Pillay et al. (2021) showed that the referral pathway for children with ASD from parental concern to accessing educational services required navigation across complicated inter-governmental service systems. Therefore, it would be important to explore and understand education services for children with ASD not only through an educational lens but also through a broader inter-governmental perspective. This study therefore set out to build on findings from Franz and colleagues (2018) on government stakeholder perspectives of ASD related services by exploring the perspectives of key government stakeholders on current ASD educational and other services in the Western Cape and potential solutions to meeting the educational needs of children with ASD and their families.

## Methods

### Design

A pragmatic qualitative research methodology (Major & Savin Baden, 2013) was used to collect and explore government stakeholder perspectives of ASD services in the Western Cape. A pragmatic research approach allowed the researcher to use interviewing as the most sensible and practical method available in order to answer the research question. Instead of having the goal of thick description (as in ethnography), theory development (grounded theory) or interpretive understanding of experience (phenomenology), the researcher aimed to discover and understand the perspectives of the people involved by providing ‘basic and fundamental qualitative descriptions’ of facts and feelings in the everyday language of the participant, as interpreted by the researcher (Sandolowski, 2000, p. 335).

### Participants and procedures

Senior level provincial government stakeholders including directors and managers were identified and recruited through purposive and snowballing sampling. As a first step, government stakeholders known to the research team were approached and recruited. Participants were then asked to recommend additional names for participation in the study and those individuals were subsequently approached directly. A total of six representatives participated in the study, two from each of the following public sector departments in the province: the Western Cape Education Department (WCED), Department of Health (DoH), and the Department of Social Development (DSD). Selection criteria included (a) knowledge about ASD services and practical experience of working with ASD at various governance and policy levels, including service planning and interdepartmental coordination of services, and (b) willingness to participate in the study. Participants were recruited telephonically or via email by the first author, and written informed consent to be interviewed was obtained. To ensure anonymity, a summary of the participant characteristics is not provided here.

### Data collection

Six individual semi-structured interviews of approximately 40–60 min each were conducted by the first author and were digitally recorded. A literature review informed the development of the interview guide which included broad, open-ended questions and clarification probes to ensure the following research questions were being answered: ‘What do government stakeholders think about the current educational services and services in general for children with ASD in the Western Cape Province?’ and ‘What do they propose for improved service delivery?’. The interviews were conducted in English and were terminated when the interviewer and interviewee felt that saturation was reached.

### Data analysis

A total of 5 h 30 min of data were yielded from the audio recorded interviews and was transcribed verbatim. NVivo version 12 was used for data storage, management and first level inductive coding to identify units of meaning expressed by participants. Second-level coding of inductively identified codes was done manually as described by Major and Savin Baden (2013) and involved grouping codes into sub-categories and categories of meaning from which an overarching theme emerged.

### Scientific rigour

The four criteria for ensuring scientific rigour in qualitative studies were applied (Lincoln & Guba, 1999). Credibility was enhanced by the primary researcher’s prolonged engagement as a therapeutic service provider in the field and familiarity with the ASD landscape in the province. This immersed positioning contributed to the development of the interview guide and the use of appropriate probes during the interviews. Researcher bias arising from immersive positioning was managed through clarifying biases with the research team, and in so doing, bracketing assumptions about ASD services in the province (Bless et al., 2013). Dependability was achieved by keeping an audit trail of the data collection and analysis processes and maintaining sustained reflexivity with the research team. The codes, sub-categories, categories and theme were agreed upon by the research team. Data triangulation involved collecting data from various sources, namely, the interviews, the first author researcher field notes and document reviews. Transferability involved the use of purposive and snowball sampling to collect a diverse range of perspectives of senior government stakeholders, thereby increasing the likelihood that the findings can be broadly generalised as representative of the perspectives held by current government officials at a particular point in time in the development of education services in a particular governance context.

### Ethical approval

Ethical approval was granted by the University of Cape Town Human Research Ethics Committee (HREC reference:072/2016) and the Western Cape Department of Education (reference: 20150727-1712).

### Community involvement

No individuals with ASD or their family members were directly involved in this study. Six government stakeholders with ASD experience at various governance and policy levels were involved.

## Results

Table 1 provides a summary of the main theme, categories and sub-categories identified in analysis. The overarching theme ‘We are doing damage control’ reflected government stakeholders’ perspectives that ASD services are falling between the ‘cracks’ created by competing societal demands on government resources and systems. At best, public sector departments were utilising available capacity in siloes to do damage control by ‘stop-gapping’ the emergent service needs of the ASD population in a reactive manner. As summarised in Table 1, participants expressed perspectives about current ASD services in three categories, each comprised of three sub-categories: (a) *A cracked society*, (b) *Siloed service systems*, and (c) *Gap-filling strategies*. In the first category, stakeholders identified contextual, governance and ideological factors as playing a role in the State’s responsiveness to the service needs of specific populations such as children with ASD. The second category summarised participants’ views of the bureaucratic, knowledge and resource barriers that obstruct proactive responsiveness by government departments to specific service demands, and in so doing, creating siloes in service delivery. The third category included perspectives on potential actions to ‘filling gaps’ that may lead to improved service delivery for children with ASD and their families.

**Table 1.** Summary of theme, categories, sub-categories and description of sub-categories.

Overarching theme ‘We are doing damage control’		
Category	Sub-category	Description of codes as meaning units in sub-category
A cracked society	Contextual factors	Aspects of the South African society that place demands on government resources and departmental service systems
	Governance factors	Perspectives on departmental responsibility and accountability for ASD services
	Ideological factors	Ideals and values informing how government stakeholders engage with societal needs
Siloed service systems	Bureaucratic barriers	Procedural obstructions at the expense of efficiency and common sense
	Knowledge barriers	Knowledge limitations about the ASD waiting list and service needs of children with ASD across government departments
	Resource barriers	Resource restrictions preventing service organisations from functioning effectively
Gap-filling strategies	Structural strategies	Arrangement of and relations between the parts or elements of the complex whole of the ASD service delivery system
	Direct intervention strategies	Practical actions, steps and service-orientated approaches
	Political strategies	Ways of navigating power dynamics between different entities

ASD: autism spectrum disorder.

The theme, categories and sub-categories and descriptions of sub-categories will be discussed in the next section. Given space limitations, a limited number of representative quotes are provided here per sub-category. Additional quotes are provided in Supplementary Material.

### Category 1: A cracked society

This category describes the external, societal factors affecting government departments’ ability to provide services for children with ASD and their families. Here, we present the sub-categories pertaining to external influences that included contextual, governance and ideological factors with representative quotes.

#### Contextual factors

Participants were of the opinion that socio-political circumstances of the country contributed to inefficiency (qualitatively described as ‘cracked’) in state departments thus restricting their ability to be proactive in planning and delivering appropriate ASD services. With government departments under pressure to meet a range of public health and social needs, the importance of contextualising the ASD situation within a larger systemic problem was raised:

… you’ve got life-threatening illnesses, you’ve got emergencies, you’ve got trauma, you’ve got violence, you’ve got a range of issues, public health issues, that we need to respond to, and we need to try to get to everybody, to have a response for everything. And it’s that context, where we find ourselves now … (P6)

#### Governance factors

Participants noted that ASD service inefficiencies and ‘cracks’ were created by lack of clarity as to the departmental ownership and responsibility for the ASD situation. Participants felt that the urgency of providing services for children with ASD was not a priority for all government departments with the Education Department being under more pressure than the health and social development departments because of the ASD education waiting list:

… the only department under pressure at the moment is Education, because of the waiting list and it can be proven. (P1)

With government being unable to provide services for children with special education needs in rural areas and informal human settlements, unregulated services by lay providers are ‘mushrooming’ in the form of day-care centres where the educational needs of children with ASD are not being optimally met. Participants noted that the support provided to these centres by the Department of Social Development was a short-term solution to a problem that required much more thought:

… but it still doesn’t mean that the school is a formal school … So I that, I think is a half-baked service … It’s a plaster that’s been put on a sore, because it is one department taking up another department’s responsibility … I think we’re really trying to heal something by quick fixes, and not thinking it through. (P4)

#### Ideological factors

This sub-category contained participants’ views about the ideals and values informing how government stakeholders engage with societal needs. Participants felt that because ASD only affected a small percentage of society, the issues that people with ASD and their families experience do not receive due attention from government:

You know, you can have community activism and you can have a swell of people talking about autism, etc., but I don’t think it’s the kind of issue that … because the numbers are so small and it doesn’t affect a large spectrum of society, it’s not the kind of issue that probably normally ends up in the general public domain … and I think, people, from politicians to government people, generally respond in terms of a big societal burden, and autism is probably not one of them yet. (P6)

### Category 2: Siloed service systems

This category addressed intra-departmental fragmentation associated with the lack of clarity about the role of different stakeholders, poor sharing of information between education, health and social development, and limited decision-making capacity. It described the internal departmental bureaucratic, knowledge and resource barriers that pose challenges for each of the government departments in meeting the needs of children with ASD and their families. These intra-departmental ‘cracks’ were viewed as a contributing factor to siloed service delivery leading to poor interdepartmental collaboration.

#### Bureaucratic barriers

Stakeholders expressed frustration with the bureaucratic barriers that influence access to information and service delivery within and between government departments. Participants acknowledged that large government departments with hierarchical structures and protocols in place posed challenges for effective decision-making and action:

… If you look at [government departments], they’re such big organisations… You need to go through three hundred channels to get a budget. You need to go through three hundred channels to do an intervention. So for me it is a pure bureaucratic structure. (P5)

#### Knowledge barriers

Government stakeholders felt that varying degrees of knowledge about the ASD service–related challenges in the province contributed to systemic dysfunction. For example health and social development government departments not having access to the Education Department’s ASD waiting list information meant that they were not aware of the extent of the situation. The lack of information about the prevalence of ASD posed challenges for planning and resource allocation:

… there’s a large unmet need that we don’t actually know about, and if we don’t know about it, we can’t plan for it. So from a resource and planning perspective, that’s actually quite an important challenge for us. It’s important for us to know what’s that burden of disease out there, that we need to be planning for, what’s the gap from what we have, and from that burden is, and how can we close that gap. (P6)

Participants felt that not knowing how to categorise and capture a diagnosis of ASD under existing government frameworks has resulted in a lack of a clearly defined focus on service delivery for these children:

… but I think the one part of all of the services being rendered to children in general, that when you do have children with specific special needs, which is not always captured very well within a government perspective. So that, it is as if the main focus would be just children in general, but forgetting, now suddenly when you get an autistic child that needs services, or a child with any other pathology, then what now? … they will say, sorry, we don’t deal with this … (P5)

#### Resource barriers

The need for more physical and human resources were highlighted. Participants felt that the lack of ASD services was a direct consequence of limited financial resources available to government departments to provide services for autistic children:

There isn’t a lot [money] going around. The country is going through a little bit of a fiscal problem at the moment. So whatever resources we have, we use it, you know, and we want to place all the children. So that’s our long term goal. (P2)

Participants felt that ASD was an expensive diagnosis to manage and children from poorer families and rural areas were more likely to have poor access to services:

A child with autism in an impoverished or poor family, is compoundedly marginalised. Because it is an expensive disability, when you are rehabilitating, when you are intervening, you know, in terms of what they need. (P1)

The lack of human resources in the form of professional and academic leadership to articulate and create knowledge and awareness about the ASD situation was highlighted:

I think our biggest gap is professional leadership. I think academic leadership, professional leadership, I think university leadership. And when you have a problem like this then you have a serious knowledge problem and a serious advocacy problem, because academics who are researching in the field, should make a case for autism. (P2)

### Category 3: Gap-filling strategies

This category describes potential actions to fill the service gaps caused by ‘societal cracks’ and ‘siloed service systems’. Structural strategies, direct intervention strategies and political strategies were identified by government stakeholders as areas to improve services for children with ASD and their families.

#### Structural strategies

Having structures in place for government departments to work collaboratively at various levels was proposed. Participants highlighted the need for a collective government strategy for addressing the service needs of children with ASD:

… and I suppose it’s not only something that you can put in front of education’s door. I think that we as government, collectively have to take responsibility for it, because if the child is identified early through the health system, what are we doing to notify the rest of the departments, in a seamless way, that the child gets picked up and seen. (P4)

#### Direct intervention strategies

Despite the numerous challenges that government departments face, participants felt that efforts to impact and improve the lives of the children with ASD and their families should not be disregarded:

There are all sorts of things, and you know, it becomes overwhelming, and then you’ve got to take a step back and say, well we’re assisting this one child, and that makes an impact on somebody’s life, and somebody’s family, and somebody, you know… (P1)

In discussing strategies to support children with ASD and their families, some participants felt that there should be intervention for children waiting for education services and others felt that children with ASD should be allowed into the education system from an earlier age. Where services were being offered, participants suggested that government departments should not duplicate services:

… if we start at three, the way we start with the deaf, there I feel we’re making a dent … it’s amazing to see how they catch up and how they can compare to their counterparts in mainstream… With deaf education, there is no waiting list at all. So if a deaf child comes into the system, he is taken in immediately. (P3)

The provision of more infrastructure or repurposing of existing infrastructure to accommodate children with ASD in the education system was proposed. Mainstream inclusion of children with special education needs, including ASD was advocated:

I still think there is not that buy-in with mainstreaming children … You’ve got the right to education, no matter what kind of child you are, or what kind of issues you have. (P5)

Participants were of the opinion that the service gap could be reduced if more financial resources were available:

… the only way that we are going to solve the waiting list is more resources. More Rands and Cents and there is less Rands and Cents. (P2)

#### Political strategies

Participants felt that finding solutions to the current situation with ASD services would require active participation from higher authorities like national policy makers and political figures:

I’ve been part of activism for different causes, and it generally always worked … But unless you can involve the decision-makers, unless you’re actually going to get active participation from decision-makers, you’re not going to get very far very quickly. (P6)

One participant referred to litigation against the South African Government for denying children with severe and profound intellectual disability the Right to Education (*Western Cape Forum for Intellectual Disability v Government of the Republic of South Africa*) and stated that there have been positive gains in service delivery in response to the court order:

I think, as much as we are often complaining, I think the court order brought us to a focus point, where we had to improve services, even though it’s not the children you are seeing. But it’s the children that’s more profound and severe, but that’s including those with autistic symptoms. (P4)

Participants felt that a ‘Whole-of-Society Approach’ could improve collaborative efforts between government departments and break down bureaucratic barriers:

Whole-of-Society Approach is aimed at trying to reduce bureaucratic obstacles that we tend to find with their collaborative efforts between government departments, and its working well, where we have piloted. We’re starting to find people who don’t normally talk to each other … people at the operational level, that they wouldn’t necessarily normally engage with each other and are able to resolve a whole host of issues. (P6)

## Summary of key recommendations from participants for strengthening ASD services

Table 2 shows a summary of the ten key strategies for ASD service strengthening in the Western Cape, which will be incorporated into the ‘Discussion’ section.

**Table 2.** Proposed strategies for ASD service strengthening in the Western Cape.

No.	Gap-filling strategies proposed
1	Formalising collaboration within and between government departments
2	Formulating a collective government strategy for ASD
3	Provision of intervention for children waiting for education services
4	Revisiting the compulsory school-going age for children with ASD to begin school at an earlier age
5	Promoting mainstream inclusion of children with ASD
6	Avoid duplication of services between government departments
7	Creating of more infrastructure or repurposing of existing infrastructure to accommodate children with ASD in the education system
8	Making more financial resources available for ASD services
9	Getting active participation from higher authorities like national policy makers and political figures
10	Adopting a ‘Whole-of-Society Approach’ for ASD to break down bureaucratic barriers

## Discussion

In this study, we explored the perspectives of government stakeholders about ASD educational and other related services in the Western Cape Province of South Africa and sought their proposals to improve services. The overarching theme ‘We are doing damage control’ suggested that government departments were not doing what they should be doing for children with ASD and their families. Government stakeholders felt that the challenges in service delivery for children with ASD were part of a greater ‘cracked society’ problem, in which the legacies of apartheid are being addressed through rigid bureaucratic government systems with limited budgets. Poverty, crime and the burden of other diseases outweigh the needs of children with ASD and their families. However, based on the South African government’s ratification of international disability policies, we argue that the State has a legal obligation to provide services for children with ASD despite competing priorities. The constitutional right to inclusive education for children with ASD is an absolute human right that cannot be neglected.

Poorly developed inclusive education systems are not unique to South Africa (Bornman & Donohue, 2014; Hodgson, 2018; Van Reenen and Combrink, 2011) with Hodgson (2018) asserting that the United Nations Convention on the Rights of Persons with Disabilities (CRPD) was developed in the global context of unsatisfactory systems for inclusion of people with disabilities. The CRPD states that every person with disabilities has the right to equal opportunities, inclusion and full participation in society. South Africa signed the CRPD in 2006 which was ratified in 2007, accepting the obligations of this legally binding instrument. However Van Reenen and Combrink (2011) argue that progress in incorporation of the principles of the CRPD in African countries, including South Africa has been slow, bordering on neglect. Ngang (2021), in examining the government’s apparent disassociation from its constitutional commitments to meeting the socio-economic rights of its people including access to healthcare, social security and education, argues that a human rights approach is central to socio-economic transformation in South Africa. The right to education for all children, including those with disabilities like ASD is enshrined in Section 29 of the Constitution of South Africa and government therefore has a legal obligation to provide timely access to education for these children. Ngang (2021) asserted that when government is unable to take necessary measures for socio-economic transformation, as stipulated in the Constitution, the Constitutional Court is obligated to intervene and ensure that the government takes action.

Pillay et al. (2022) provided a factual reflection of the education system’s failure in policy enactment. They reported that despite having progressive national education policies in place to support the inclusion of children with disabilities in the education system, a large number of children with ASD in the Western Cape Province are either out of school, awaiting school placement or attending over-subscribed special ASD schools. Hodgson (2018) described South African inclusive education policies as ‘nice theories’ developed by academics and activists to correct historic injustices, but argued that theories alone are not enough for disability inclusive transformation. Bornman and Donohue (2014) agreed that ambiguous policies born out of political reason are often passed down with little accountability for implementation.

Poor policy implementation is not exclusive to the education system. Franz and colleagues (2018) reported that even though the principles of National Integrated Early Childhood Development (ECD) Policy (South African Department of Social Development, 2015) supported early detection and intervention for children with ASD, there was poor implementation of these policies for children with disabilities in general. Mokitimi and colleagues (2018) found that there was concerning neglect of Child and Adolescent Mental Health (CAMH) policies despite the recognition of the associated burden of CAMH in South Africa.

Government stakeholders in this study attributed the lack of policy enactment evidenced by poor service delivery for children with ASD in part to a lack of knowledge of the extent of the ASD burden and to difficulties with capturing the diagnosis under existing government data frameworks. Franz and colleagues (2018) reported similar difficulties with categorising the diagnosis finding that children with ASD are included under the Department of Health’s data framework for intellectual disability with implication for shared funding and resource allocation. Baxter et al. (2015) state that accurate epidemiological description of ASD is imperative for policy development and planning. Epidemiological studies on ASD are not feasible in LMIC like South Africa for various reasons including a lack of culturally and linguistically appropriate assessment tools (de Vries, 2016; Pillay et al., 2021; Smith et al., 2017). Despite the lack of epidemiological data, we however argue that there is substantial evidence of education systems failure to provide services for children with disabilities in South Africa for the government to act (Hodgson, 2018; Human Right Watch, 2015; Ngwena, 2013).

One of the challenges in service delivery for children with ASD described in this study was the poor communication and collaboration within and between the different government departments. Orsini and Smith (2010) examined the interaction between social movements, knowledge mobilisation and policy for individuals with ASD and argue that the process should not be a ‘top-down’ approach from government and instead be a collaborative and interactive effort between government and civil society to create knowledge. Lord et al. (2022) suggest that coordination between government sectors, including individuals with ASD and their families is essential for meeting the complex needs of these individuals and their families. The recurring theme of government ‘silos’ in South African studies (Franz et al., 2018; Mokitimi et al., 2018) confirms that government departments need to develop systems for working together. Having structures in place to formalise collaboration at various levels instead of working in silos and duplicating services would be in line with the WHA 67.8 resolution (WHO, 2014) which recommends that government ‘strengthen different levels of infrastructure for comprehensive management of autism spectrum disorders and other developmental disorders’ (WHO, 2014).

Government stakeholders in this study felt that the lack of finance was a significant barrier to providing services for children with ASD. Hodgson (2018) asserted that the lack of financial resources cannot be an excuse for not delivering on human rights obligations and argues that existing funds have been misused by building more special schools instead of promoting the inclusion of children with special learning needs in existing mainstream and full service schools. The WHA 67.8 resolution recommends that governments facilitate resource mobilisation especially in countries with limited resources to address the needs of individuals with ASD (WHO, 2014). According to Mayosi and Benatar (2014), there should be a shift in attitude in government to incorporate a ‘doing better with less’ philosophy in light of the limited available resources and poor prospects of economic growth in the country.

One way of using the limited resources more economically would be to explore various educational settings for children with ASD and not rely almost exclusively on special education placements which are more costly for governments than ordinary mainstream schools (UNICEF South Africa, 2018). In 2016, the annual cost of educating a single child in the special education system in South Africa was approximately R57,000 per annum (~US$3983.29) which is four times more than the cost of educating a child in mainstream schools (UNICEF South Africa, 2018). Advocacy and awareness campaigns to promote ASD inclusion in mainstream schools and ECD centres as well as resource mobilisation to strengthen these systems to accommodate children with ASD from as early as possible is recommended.

Participants in this study highlighted that the government generally responds to big societal burdens and because ASD only affected a small portion of society, the challenges that individuals with ASD and their families experience often do not receive enough attention from the state. However, participants also drew on the example of consumer led activism where caregivers of children with severe and profound intellectual disability (CSPID) sought litigation against the South African Government through the Western Cape Forum for Intellectual Disability (WCFID) for denying these children the Right to Education (Murungi, 2011). The court case forced government departments to work together to develop a strategy to meet the courts mandate and improve services for children with severe and profound intellectual disabilities (McKenzie et al., 2017). Similarly, developing a strategy to meet the needs of children with ASD and their families would require active participation from authorities like national policy makers and political figures.

South Africa became a signatory to the United Nations 2030 Agenda for Sustainable Development that makes a commitment to ‘leave no one behind’. The Western Cape Government’s commitment to the ‘Whole of Society Approach’ has been operationalised by putting health and safety projects in place to improve the lives of all of its citizens (Western Cape Government, 2015). Leigh and Du (2015) predicted that by 2025 the cost of ASD on society will far exceed that of diabetes and attention deficit/hyperactivity disorder (ADHD) if the numbers of ASD continue to increase as it has in the past. In light of the growing burden of ASD similar projects for children with ASD and their families are indicated through engagement with government, civil society, private sector and media.

Government stakeholders highlighted numerous systems challenges in delivering appropriate and equitable services to children with ASD. They also provided 10 gap-filling strategies that, if actioned, could go a long way to improving services for children with ASD and their families in the Western Cape as well as other parts of South Africa.

### Limitations

We acknowledge that senior-level government stakeholders from the three main government departments in a local context is by nature a small group of people. While the small sample size in the study may be seen as a limitation, it nevertheless provides a representative perspective of persons concerned with high-level governance of ASD services. Second, we collected data from government stakeholders only in the Western Cape, and acknowledge that their perspectives may not be representative of the situation in other South African provinces. However, given that the Western Cape is frequently regarded as one of the better resourced provinces for children with ASD in South Africa, we can assume that the situation in some of the other provinces in the country could be far worse. Further research would be necessary to evaluate government perspectives on ASD education and clinical services in the rest of the country and other LMIC.

### Relevance to other low- and middle-income countries (LMIC)

Even though this study explored the perspectives of government stakeholders about educational and other services for children with autism in one province of South Africa only, we propose that our findings may have relevance for and resonance also with other LMIC contexts. Knowledge and research evidence about autism is generally very low in LMICs (Franz et al., 2017; Hahler & Elsabbagh, 2015; Lord et al., 2022) and government departments and policy makers in most LMICs do not prioritise ASD research and service delivery (Hahler & Elsabbagh, 2015). Therefore, insufficient national funds are allocated to meet the needs of children and adolescents with neurodevelopmental disabilities such as ASD in LMICs (World Health Organization, 2018). The lack of trained mental health professionals, leading to gaps in identification and support for those with autism, is another theme from our work that is very similar to the majority of other LMICs, as are the disparities in service delivery between urban and rural communities (Divan et al., 2021; Lord et al., 2022). For these reasons, we believe that many of the ‘gap-filling strategies’ recommended by participants in this study, may be of value also to other LMICs.

## Conclusion

Qualitative insights into the ‘software’ elements of government values, norms and relationships that inform ASD service delivery in the Western Cape contributed towards a ‘whole systems’ understanding. Despite having progressive inclusive education policies in place, many children with ASD are out of schools. The education system’s failure in policy enactment for children with ASD is concerning. Fast-tracking policy translation into practice so that all children with disabilities can be accommodated in the education system as early as possible should be a priority of government. Mobilising resources and using existing resources more optimally to create educational opportunities for children with ASD is proposed. Collaboration between government departments and other relevant stakeholder groups to develop a collective strategy for ASD in line with the WHA67.8, ‘Comprehensive and coordinated efforts for management of autism spectrum disorder’ is recommended.

## Supplemental Material

sj-docx-1-aut-10.1177_13623613221142111 – Supplemental material for ‘We are doing damage control’: Government stakeholder perspectives of educational and other services for children with autism spectrum disorder in South Africa Click here for additional data file. Supplemental material, sj-docx-1-aut-10.1177_13623613221142111 for ‘We are doing damage control’: Government stakeholder perspectives of educational and other services for children with autism spectrum disorder in South Africa by Sarosha Pillay, Madeleine Duncan and Petrus J de Vries in Autism
