# Supplementary material for: ‘We are doing damage control’: Government stakeholder perspectives of educational and other services for children with autism spectrum disorder in South Africa
Source: Autism. 2022 Dec 13;28(1):73–83. doi: 10.1177/13623613221142111 (PMC10771016; doi:10.1177/13623613221142111)
Supplement: sj-docx-1-aut-10.1177_13623613221142111 – Supplemental material for ‘We are doing damage control’: Government stakeholder perspectives of educational and other services for children with autism spectrum disorder in South Africa [file sj-docx-1-aut-10.1177_13623613221142111.docx]

**Supplementary material**

| **Additional quotes from government stakeholders** | | |
| --- | --- | --- |
| **Category** | **Sub-category** | **Quotes** |
| A cracked society | Contextual factors | *“This country, is in such a bad space, economically, psychologically…If you see how people think its ok to steal; if you see how many people are on drugs; so many people are unemployed, in poverty; so all of that leads to…all those different variables, that leads to these kinds of things happening…and that’s the bigger picture.” (P5)*  *“I think that it is important to contextualise, because if you look at autism specifically, then you will obviously understand why…maybe put some context to why the impression could be that we’re not taking it seriously enough. And we’re not…by that I mean that we…and I’m using the resource allocation as a proxy, for how seriously we’re taking it. So when you look at the funding that’s available for autism, you might actually think that we’re not...” (P6)* |
|  | Governance factors | *“…who is ultimately responsible for these children?” (P2 )*  *“I feel that [government department] does not play their role, because children from two years, three years to five years, its actually their problem…their responsibility” (P3)*  *“…many of these centres start off with a mummy in either a rural setting, or in informal settlements…she takes anyone with disabilities into the day care centre…so you’ve got this combination of different levels of functioning…” (P4)*  *“…and because of the resources in the community, that the only place where ‘special children’ go…it becomes an organic way of including the children so that the parents can go and work.” (P1)* |
| Siloed service systems | Bureaucratic barriers | *“In [government department] where I’m sitting, I don’t have any decision-making authority. In the Western Cape, there is a clear line between services and support, and we fall under support and the services do the planning, make the decisions and implement. So there’s at the moment, a huge fragmentation... You go to national, you’re not sure what’s happening in the department, we have to scout for information. So I think that is a big thing” (P4)*  *“..the wheels of government do turn slowly, and I’m speaking as an advocate, not as a departmental person. So the wheels do turn…and sometimes frustratingly so. And as that happens, we can say, yes we’ve ticked the box on certain things, and we’ve grown a service, but it only actually…it only scrapes off the tip of the iceberg.” (P1)* |
|  | Knowledge barriers | *“I’m not aware where the waiting list is standing at the moment. I do know that there is a very slow entrance – if I can call it that – because of mainly complaints coming from the Human Rights Commission, on access to schools. But where it is at the moment, that would be interesting.” (P4)*  *“The one sad part for me, is that this is now again a stumbling block, the Education Department’s waiting list system, we’re not allowed to have access to that system. I still don’t have a clear answer why… we’ve been asking for years, to get access to it, so if we’ve got kids in our places, we can put them on the list.” (P5)*  *“So ASD has historically fell under intellectual disability, which may not be quite the right place to put it, because we know we’ve got different levels on the spectrum and not everybody falls under intellectual disability. But I think that there are silos that the department used to work in, so for example, HIV and TB, mental health, etc. and it was hard to place a person not in that particular silo. So there wasn’t anything for ASD. The closest it could align with was, because we so many ASD children are seen in the intellectual disability framework, and therefore because it was seen as being under the Mental Health Care Act, it’s currently located there…historically there is also a funding stream, that was decided on and depending on the funding, that department is responsible for the service” (P4)* |
|  | Resource barriers | *“Where there’s money, you see pretty much a well-run programme. That same child… put that same child into a different scenario, and you will see the same child sitting there and rocking and doing very little.” (P4)*  *“If there was enough space and services then there wouldn’t be that waiting list.” (P4)* |
| Gap-filling strategies | Structural strategies | *“…where those people control those resources, need to work more closely together. And it’s difficult, because it’s across government departments, that it comes with challenges. It’s not that system has been set up for that to happen more structurally.” (P6)*  *“…we need to have collaboration at a more operational level…In other words, people who actually deal with the… besides the heads of departments, at a senior level, they’ll need to sign the framework agreements – if I can call it that – an operational level, we need to work out which patients need to be placed where. How do we reduce the waiting lists? How do we impact peoples’ lives at that level?” (P6)*  *“We haven’t, in any conversation that I’m aware of, departmentally. government as a whole in the Western Cape, has not focused on ASD per say; it’s always included in something else…if you look at the inter-government forum, a strategy has never been from the District General’s office to say…what is our strategy for people with ASD? Education are you taking full responsibility? Health are you covered at all levels of care? Will people deal with it? This hasn’t been discussed to my knowledge, not in the time that I’ve been here, and I’ve been in post for about seventeen years.” (P5)* |
|  | Direct intervention strategies | *“I think that the waiting list should not be something stagnant. There should always be some intervention for the waiting list kids…I really think that such interventions should be taken up by the schools. But also, you know, if you don’t drive it from one central point, that’s not going to happen. So I would like us, in the system to go and sit and look at one model, that we can give out to schools and say, start with this. And there also post provision would play a role. If we want that, we will have to create a post for a person to do that throughout the week.” (P3)*  *“If I look at infrastructure also, upgrading infrastructure, if I look at children in special needs care. I want part of the solution to be the special care centres that’s mushrooming up in the communities must be part of a mainstream school close to them, not necessarily an LSEN School (Schools for Learners with Special Education Needs). In other words, if I can get classes built on the side of the school, but they are part of the school set-up, on the premises. That’s what I’m moving towards, that’s the vision I have…” (P5)*  *“Something that I also feel is going to make a big dent [in the waiting list numbers], is the satellites that we are starting, where stronger schools will have satellites from which they can operate, they can drive the satellite from the school side and have more classes there.” (P3)*  *“And what we are trying to say, is that if you are rendering a physio service there, then don’t let the Education team come in and do the same. And the same for Health, we’ve got OT’s, Physios, not a lot of them, but we also do outreach. So what roles…it’s defining the roles and responsibilities. And I think because the meetings were stopped, there wasn’t a space for people to actually say, guys, this is what we’re doing, why are you doing the same thing? Or why are we not doing anything?” (P4)* |
|  | Political strategies | *“…We probably need a focal point on autism, at that level, to start off there… these are the Ministers of Health, Heads of Departments, these are the big shots, these are the higher more senior people… if we had at that level, a focus intervention at that level on autism, I think that’s a start. Because with a focus intervention like that, would come departmental programmes, on dealing with autism; so Health, Education, Social Services, would be the lead departments…if we do that in collaboration, then I think we would be able to get somewhere. Now at that level of government, the Premier is able to influence programmes or whatever the Premier decides on, and commits resources to.” (P6)*  *“…we live within the context of the legislative parameters that we have, and so we do have certain protocols…but I think if we had a transversal approach, programmatic response to autism, across government departments, you know, if policy is issued around that, then we would be able to, I think more clearly articulate a collective government response to autism, to children with autistic disorder, and adults, for that matter, to pick them up and help.” (P6)* |
